# Starting off on the right foot: strong right-footers respond faster with the right foot to positive words and with the left foot to negative words

**DOI:** 10.3389/fpsyg.2015.00292

**Published:** 2015-03-20

**Authors:** Irmgard de la Vega, Julia Graebe, Leonie Härtner, Carolin Dudschig, Barbara Kaup

**Affiliations:** Department of Psychology, Language and Cognition, University of TübingenTübingen, Germany

**Keywords:** embodiment, body-specificity hypothesis, handedness, footedness, emotional valence, fluency

## Abstract

Recent studies have provided evidence for an association between valence and left/right modulated by handedness, which is predicted by the body-specificity hypothesis (Casasanto, 2009) and also reflected in response times. We investigated whether such a response facilitation can also be observed with foot responses. Right-footed participants classified positive and negative words according to their valence by pressing a key with their left or right foot. A significant interaction between valence and foot only emerged in the by-items analysis. However, when dividing participants into two groups depending on the strength of their footedness, an interaction between valence and left/right was observed for strong right-footers, who responded faster with the right foot to positive words, and with the left foot to negative words. No interaction emerged for weak right-footers. The results strongly support the assumption that fluency lies at the core of the association between valence and left/right.

## Introduction

Recent research has provided evidence for an association between positive/negative and left/right. In a series of studies, Casasanto (2009; see also Casasanto and Chrysikou, 2011, and Casasanto and Henetz, 2012) showed that positive valence is associated with the dominant hand or side, and negative valence with the non-dominant hand. For example, right- and left-handers were presented pairs of novel objects, one located on the right side, one located on the left, and had to decide which of these objects was more attractive or happier. Results showed that handedness influenced this decision: right-handers tended to ascribe these positive characteristics to the object located on the right, whereas for left-handers, the opposite pattern emerged: they tended to choose the object on the left for these positive characteristics. This association between valence and left/right is also reflected in response times: when participants classify positive and negative words according to their valence, right-handers respond faster to positive words with their right hand compared to their left hand, and faster to negative words with their left hand compared to their right hand. Left-handers, on the other hand, show the opposite pattern: they respond faster with their left hand to positive stimuli, and with their right hand to negative stimuli (de la Vega et al., 2012). This pattern – faster responses to positive items with the dominant hand, faster responses to negative words with the non-dominant hand – even shows when participants hold their hands crossed (de la Vega et al., 2013). Thus, it appears that the interaction between valence and left/right has its origin in different experiences individuals make with regard to their hands, and is therefore also modulated by handedness.

How does this association between valence and left/right emerge? A plausible assumption is that motor fluency – the ease with which an action is performed (see Oppenheimer, 2008) – lies at the core of this association. The dominant hand, used for the vast majority of manual actions in everyday life, such as writing or using a knife, is, of course, much more fluent than the non-dominant hand. Motor fluency is therefore directly associated with the dominant hand. A high degree of fluency, on the other hand, is associated with positive affect (see Reber et al., 1998; Winkielman and Cacioppo, 2001; Winkielman et al., 2003; Reber et al., 2004; Beilock and Holt, 2007; Oppenheimer, 2008). It seems that motor fluency serves as link between positive valence and dominant hand, and negative valence and non-dominant hand (see also Casasanto and Chrysikou, 2011; see Milhau et al., 2014, for the influence of situated motor fluency on the interaction between valence and left/right). This impact of everyday experiences of individuals in their physical environment, reflected in the association between valence and left/right, is in line with the body-specificity hypothesis (Casasanto, 2009), which postulates that mental representations shaped through interactions with the physical environment should differ for individuals with different bodies and, consequently, different experiences (see also Willems et al., 2009, 2010; Casasanto and Jasmin, 2010; Brookshire and Casasanto, 2012; Brunyé et al., 2012). With respect to its theoretical orientation and underlying assumptions, the body-specificity hypothesis can be embedded in theories of embodied cognition (e.g., Glenberg, 1997, 2010; Barsalou, 1999, 2008; Glenberg and Kaschak, 2002; Barsalou et al., 2003; Zwaan, 2004; Zwaan and Madden, 2005; Fischer and Zwaan, 2008).

Of course, most individuals do not only have a dominant hand, but also a dominant foot. Although the difference between the different degrees of motor fluency of left and right foot does not necessarily come to attention in everyday life as often as the different degrees of fluency of left and right hand, there are situations when footedness becomes relevant. For example, any hardcore football fan – or at least any coach worth his money – knows that when it comes to football, footedness matters. Most football players prefer one foot to kick the ball; for example, it is well known among fans that Diego Armando Maradona scored his goals mostly with the left foot (and once with the help of his left hand; Gutwinski et al., 2011), and a player managing to score a goal with his “wrong” foot earns often jubilant praise from coach, teammates, and commentator. Interestingly, while being left-handed is still seen as a disadvantage in certain cultures, being left-footed can in football even have advantages (see Porac and Coren, 1981; see also McMorris and Colenso, 1996; Baumann et al., 2011;Memmert et al., 2013).

Of course, there are many other situations in everyday life where footedness plays a role, although we may not always be aware of it. For example, footedness determines which foot we use when we step on a chair, or which foot we use as front foot when snowboarding. However, in contrast to handedness, the notion of the preferred foot is not always clear (Gabbard and Hart, 1996). Peters (1988; see also Gabbard and Hart, 1996) defines as preferred foot the foot used to interact with an object (e.g., kicking a ball; picking up a pebble) or the one that leads out (e.g., when jumping or when stepping up on a chair). The non-preferred foot supports the preferred foot and stabilizes its activities.

In analogy to handedness, most individuals prefer their right foot for motor actions such as kicking a ball (Coren and Porac, 1980; Gabbard and Iteya, 1996). This bias toward the right side, however, is more pronounced in handedness (around 90%; Peters et al., 2006) than in footedness (around 80%; Porac and Coren, 1981). There is evidence that footedness is a better predictor of cerebral lateralization than handedness (Elias et al., 1998), which has be attributed to less social pressure when using the left foot in comparison to left hand (Chapman et al., 1987; see also Meng, 2007, and Tran et al., 2014). Interestingly, although research indicates that for most individuals, lateral preference is the same for hand and foot, there is, in fact, an important difference between right- and left-handers. Peters and Durding (1979), for example, found that of 56 right-handers, 95% preferred their right foot for kicking a ball, whereas of 56 left-handers, only 50% preferred their left foot for this task (Chapman et al., 1987, report similar numbers when measuring the foot preference of left- and right-handers on their 11-item foot preference inventory).

Moreover, foot preference seems to undergo a shift during childhood. Gentry and Gabbard (1995) noted that whereas 26% of 4- and 8-years-olds showed a mixed foot preference, a significant shift toward a preference of the right foot was observed for children from the age of 8 to the age of 11 years: the prevalence of right-footedness for 4- and 8-years-olds, 66%, did differ significantly from the prevalence of right-footedness for 11- to 20-years-olds, 81%. There was no significant difference between 4- and 8-years-olds, nor between the different groups between 11 and 20 years, indicating that after the age of 11, no major shift of footedness preference occurs (see also Gabbard and Iteya, 1996, for a review of the data indicating prevalence of footedness and handedness in children and adults, and Porac, 1996).

In the study described here, we investigated whether the motor fluency of the dominant foot influences – in analogy to the fluency of the dominant hand – the association between valence and left/right. In contrast to handedness, footedness does not play a role in everyday life for most individuals, or only marginally. Moreover, the difference in fluency between dominant and non-dominant foot should be much smaller than the one between dominant and non-dominant hand, given that for many actions such as walking or running, both feet are used. Additionally, in everyday life and in contrast to manual actions such as writing or sewing, we usually do not perform any fine motor actions with our feet. In spite of these differences between manual actions and motor actions performed with a foot, most people should show a difference between their dominant and non-dominant foot with respect to the degree of fluency, although this difference might be pronounced less than the one between the dominant and non-dominant hand. According to the body-specificity hypothesis (Casasanto, 2009), this difference should then lead to an association of the dominant foot and positive affect.

To assess the potential association between dominant / non-dominant foot and positive / negative valence, we followed the procedure reported in de la Vega et al. (2012, Experiments 2 and 3), with the difference that participants used their feet to respond instead of their hands. We presented positive and negative words to participants, who classified them according to their valence. They pressed a key with their right foot in response to positive words and with their left foot in response to negative words, or the other way around (response with the right foot to negative words, response with the left foot to positive words). If the dominant foot is associated with positive affect due to its greater degree of fluency, responses with the dominant foot should be faster for positive words, and with the non-dominant foot to negative words, in analogy to previous findings concerning manual responses (de la Vega et al., 2012, 2013). We decided to assess in this first study only right-footers, although the pattern emerging for their dominant vs. non-dominant foot should be the same for left-handers’ dominant vs. non-dominant foot.

## Experiment

### Method

#### Participants

All participants gave informed consent. 40 volunteers participated in the experiment. Only right-footed participants were included in the analyses, reducing the total number of participants to 37 (9 male). Mean age of participants was 23.2 years (*SD* = 2.7). All remaining participants were native German speakers and right-handers, according to a translated version of the Edinburgh Handedness Inventory (Oldfield, 1971; *M*_handedness score_ = 83.68; *SD* = 18.24). We assessed footedness with a self-constructed questionnaire adapted from Chapman et al. (1987). This questionnaire contained the adapted five items for assessing footedness with the highest validity, as described in Chapman et al. (1987). Participants indicated their preferred foot for the following actions: try to kick a ball into a basket; write your name in sand; after writing the name, smooth the sand; roll a golf ball around a printed circle as rapidly as possible; kick as high as possible on a wall. Participants indicated their preference by ticking a box on the left, on the right or – in the case they did not prefer a foot – by leaving them blank. A ticked box on the right counted as 1, a ticked box on the left as -1. Participants could therefore obtain a footedness score between -5 (strong left-footer) and +5 (strong right-footer). The mean score obtained in this footedness inventory was 4.27 (*SD* = 0.99).

#### Materials and Apparatus

The material and procedure employed during the response time study were the same as in Experiments 2 and 3 in de la Vega et al. (2012), with the difference that participants responded with their feet instead with their hands. Fourty German words (20 positive, 20 negative) were used. Fourty German pseudowords served as fillers. The words were matched with regard to their frequency, but not with regard to arousal (for details, see de la Vega et al., 2012). Responses were collected with the help of a computer keyboard placed on the floor. The keyboard had a self-constructed overlay with one key on the left, and one key on the right. Participants pressed with their right foot the right key (the key END), and with their left foot the left key (the key TAB).

#### Procedure and Design

Right before the response time study, participants were asked to dribble a small ball around obstacles (plastic bottles filled with water). They did this first with one foot, afterward with the other foot, to get a feeling which foot they might prefer. After having done this, they indicated their foot preference and filled out the translated version of the Edinburgh Handedness Inventory (Oldfield, 1971) and the footedness questionnaire.

Each trial started with a fixation cross appearing centrally for 400 ms. Afterward, the item was presented for 2000 ms. Participants were asked to respond during this period. A blank screen was then shown for 1000 ms during experimental trials; during practice trials, feedback was shown during 1500 ms.

Half of the participants started by responding with their right foot for positive words, and with their left foot to negative words. In the second part of the experiment, this stimulus-response assignment was reversed. For the other half of participants, this order was the other way around. Participants did not respond to pseudowords. The same set of stimuli was used in both parts of the experiment, resulting in a total of 160 trials. Each part of the experiment started with 20 practice trials.

### Results

Incorrect responses were discarded (4.0% of all Go-Trials). RTs of correct responses were submitted to two 2 (valence: positive vs. negative) × 2 (response foot: left vs. right) ANOVAs. One ANOVA treated participants as the random factor (*F_1_*), and one ANOVA treated items as the random factor (*F*_2_).

**FIGURE 1:**
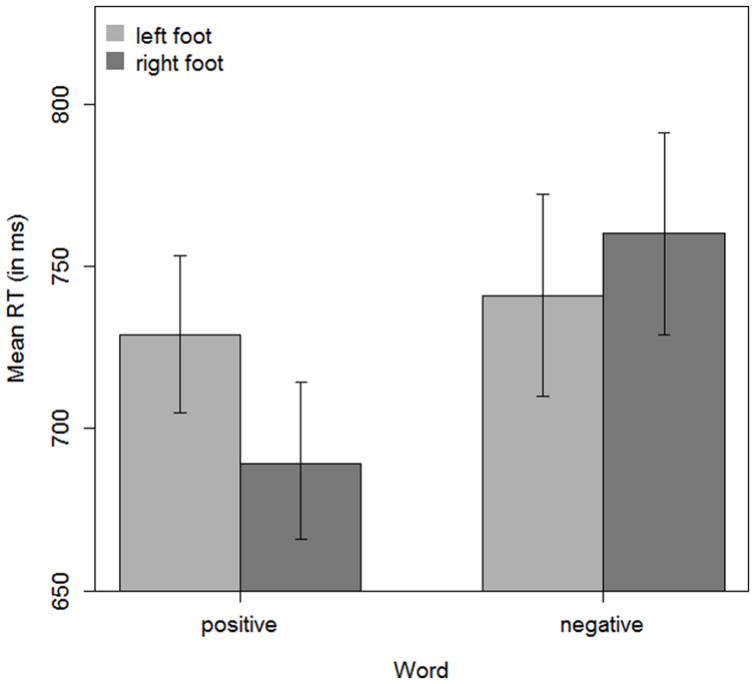
**Mean response times for all participants (*n* = 37) for responses to positive and negative words with the right foot or with the left foot.** The error bars represent confidence intervals for within-subject designs and were computed as recommended by Masson and Loftus (2003).

Overall mean was 730 ms. As in earlier studies employing the same stimulus material (de la Vega et al., 2012, 2013), a main effect for valence emerged [*F*_1_(1,36) = 39.11, *p* < 0.001; *F*_2_(1, 38) = 9.97, *p* = 0.003], with faster responses to positive in comparison to negative words (709 ms vs. 750 ms). Responses with the right foot were numerically faster than responses with the left foot (725 vs. 735 ms); however, this difference was only significant in the by-items analysis [*F*_1_(1,36) = 1.89, *p* = 0.18; *F*_2_(1,38) = 4.45, *p* = 0.04]. Crucially, although responses to positive words were faster with the right foot than with the left foot (690 ms vs. 729 ms), and responses to negative words were faster with the left foot than with the right foot (741 ms vs. 760 ms; see **Figure 1**), this difference was only highly significant in the by-items analysis, but not in the analysis across participants [*F*_1_(1,36) = 2.69, *p* = 0.11; *F*_2_(1, 38) = 24.75, *p* < 0.001].

The fact that the by-items analysis shows divergent results from the by-participants analysis might indicate that some, but not all, of the participants show the expected pattern. As the association between footedness and positive valence should depend on the degree of fluency individuals have made with their dominant foot, we decided to split the participants into two groups depending on their fluency with the right foot to further explore the data. We classified participants as “strong” and “weak” right-footers. Strong right-footers included all participants who had indicated to use only their right foot in all five questions of the footedness questionnaire (21 participants; mean score = 5.00, *SD* = 0.0); weak right-footers included the rest (16 participants; mean score = 3.31, *SD* = 0.79). The difference between the footedness scores of these group was significant [*t*(35) = 9.79, *p* < 0.001], while the difference between handedness scores was not [*t*(35) = 1.24, *p* = 0.22].

**FIGURE 2:**
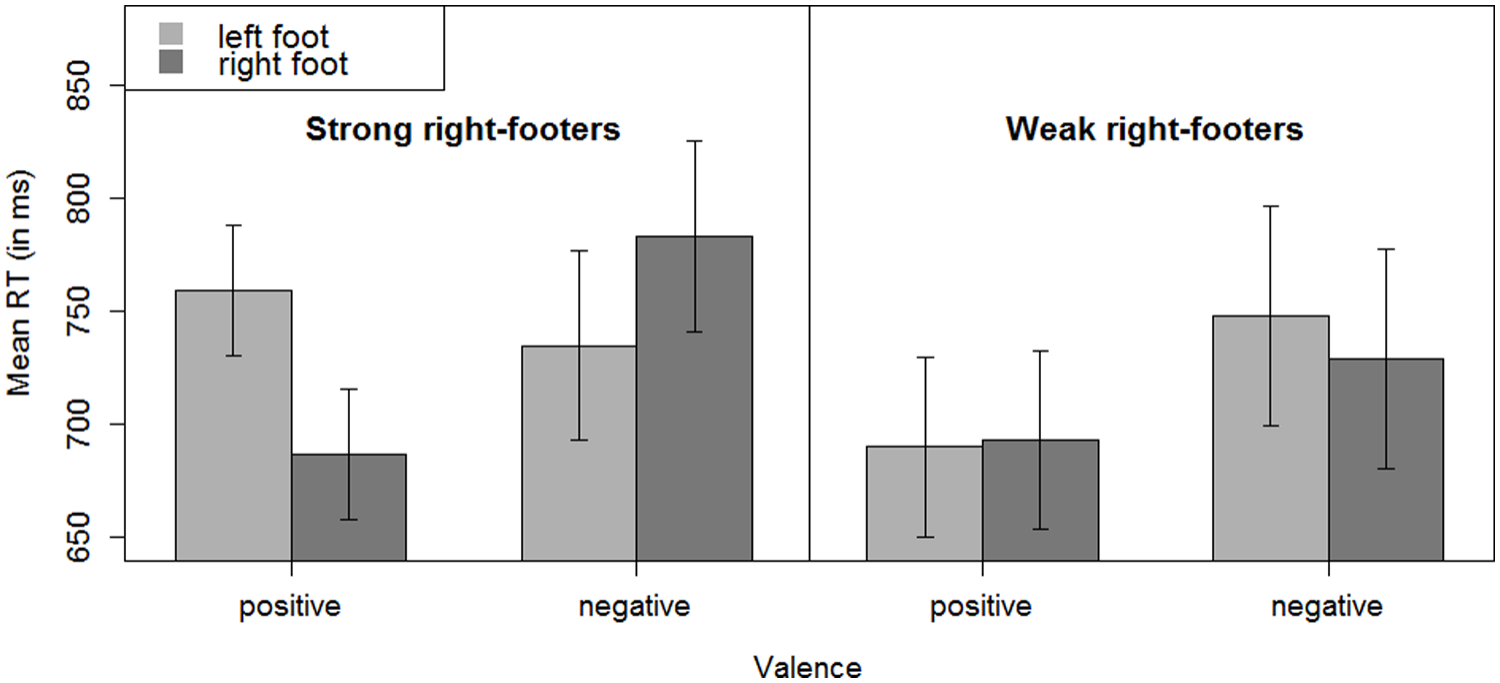
**Mean response times for strong and weak right-footers. (Left)** mean response times for strong right-footers (*n* = 21) for responses to positive and negative words with the right foot or with the left foot. **(Right)** mean response times for weak right-footers (*n* = 16) for responses to positive and negative words with the right foot or with the left foot. The error bars represent confidence intervals for within-subject designs and were computed as recommended by Masson and Loftus (2003).

We conducted the same analyses as for the whole set of participants for strong right-footers and for weak right-footers. For strong right-footers, a main effect of valence showed [*F*_1_(1,20) = 19.88, *p* < 0.001; *F*_2_(1, 38) = 5.30, *p* = 0.03], with faster responses to positive in comparison to negative words (723 vs. 759 ms). There was no main effect of foot used to respond [*F*_1_(1,20) = 1.18, *p* = 0.29; *F*_2_(1, 38) = 3.87, *p* = 0.06]. Most important for our research question, an interaction between foot and valence emerged for the strong right-footed participants [*F*_1_(1, 20) = 7.49, *p* = 0.01; *F_2_*(1,38) = 66.29, *p* < 0.001; see **Figure 2**]. We conducted separate analyses for positive words and negative words only. Faster responses showed with the right foot vs. the left foot to positive words [687 ms vs. 759 ms; *F*_1_(1,20) = 13.43, *p* = 0.002; *F*_2_(1, 19) = 35.03, *p* < 0.001]. For negative words, responses were faster with the left foot in comparison to the right foot (735 ms vs. 783 ms). However, this difference was only significant in the by-items analyses [*F*_1_(1,20) = 2.83, *p* = 0.11; *F*_2_(1, 38) = 35.21, *p* < 0.001]. Interestingly, the weak right-footers showed a different pattern: they showed a main effect for valence [*F*_1_(1,15) = 18.92, *p* < 0.001; *F_2_*(1,38) = 12.19, *p* = 0.001], with positive words eliciting faster responses than negative words, and no effect for foot used for response [*F*_1_ < 1; *F_2_*(1,38) = 1.81, *p* = 0.19]. However, they did not show an interaction between dominant foot and valence [*F*_1_ < 1; *F*_2_(1,38) = 2.66, *p* = 0.11; see also **Figure 2**].^1^ This different pattern was further corroborated when we included the strength of right-footedness as a factor and conducted an additional analysis across all participants, as a three-way interaction between valence (positive vs. negative), response foot (left vs. right), and strength of right-footedness (weak vs. strong) emerged [*F*_1_(1,35) = 4.16, *p* = 0.049; *F*_2_(1,38) = 68.39, *p* < 0.001]. Additionally, we correlated the compatibility effect of each participant (calculated by the difference between the mean response time in the incongruent condition and the mean response time in the congruent condition) with his or her footedness score. A moderate correlation showed (*r* = 0.39, *p* = 0.017). It should be noted, however, that the correlation of difference RT scores can easily lead one astray (see Miller and Ulrich, 2013) and has to be interpreted with caution, as the observed correlation does not necessarily show the same pattern as the underlying correlation.

## Discussion

Previous studies have found evidence for an association between dominant hand and positive valence, presumably based on the high degree of fluency of the dominant hand (Casasanto, 2009; Casasanto and Chrysikou, 2011; Casasanto and Henetz, 2012; de la Vega et al., 2012, 2013). If fluency indeed lies at the core of such an association, then it is plausible to expect a link between the dominant foot and positive affect – albeit maybe, due to the fact that the difference between dominant and non-dominant foot is far from being as marked as the one between dominant and non-dominant hand, not as strong as the one found between hands and valence.

We investigated the question whether people associate positive with their dominant foot in a response times study. Right-footed participants classified positive and negative words according to their valence, responding with their right foot to positive and with their left foot to negative, or the other way around. Results showed only a significant interaction between valence and foot in the by-items analysis when looking at the whole set of participants. However, when dividing participants into two groups according to the strength of their footedness, strong right-footers showed an interaction between valence and left/right foot, whereas no interaction emerged for weak right-footers.

The findings are in line with the body-specificity hypothesis, according to which the physical experiences individuals make with their individual physical characteristics in their environment should have an impact on their mental representations, such as the association between valence and left/right. Furthermore, the difference between strong and weak right-footers strongly supports the assumption that motor fluency lies at the core of the association between valence and left/right. To our knowledge, this study is the first to provide evidence that the strength of preference of one limb might have a direct impact on this association. This finding implies that an association between valence and left/right foot only comes into existence when the degree of fluency of the dominant foot is strong enough, or when the difference in fluency between left and right foot is large enough. Although we investigated only right-footers in the experiment presented here, we expect the same pattern (an association between dominant foot and positive valence, and non-dominant foot and negative valence) to show also for strong left-footers. Future studies are needed to corroborate this assumption. An interesting question in this regard is whether this pattern can also be observed for handedness: do strong right-handers exhibit an association between valence and left/right, whereas weak right-handers do not? Or can such an association always be observed, independent of the strength of handedness? Interestingly, a recent study by Huber et al. (2014) points toward the possibility that in certain cases, the degree of handedness does play a role, as a reversed MARC effect showed for stronger left-handers, but not for weaker left-handers.

If the pattern observed here is confirmed in subsequent studies investigating, for example, manual responses, the results would be difficult to reconcile with what has be considered up to now an alternative explanation for the valence-by-left/right association as found in response time tasks, namely the polarity correspondence principle (Proctor and Cho, 2006; see also Lakens, 2011, 2012). According to this principle, stimulus categories and response categories are both coded binarily, with the salient category receiving a +, the non-salient category receiving a -. It is assumed that when two categories match in the sense that they both receive a +, or that they both receive a -, response times should be faster. For the categories valence and left/right, it might be assumed that positive and right (for right-handers or right-footers) are the salient dimensions. Negative and left (for right-handers and right-footers) should be the non-salient dimensions. In a response times study, faster responses with the right to positive and with the left to negative might then be explained on the basis of a match with regard to the coding of the dimensions (see also de la Vega et al., 2013). However, this alternative explanation is difficult to reconcile with the pattern observed here: if we always code different dimensions of categories binarily according to their salience, it should not matter if someone is a strong or a weak right-hander or right-footer; as soon as the right hand or foot is stronger than the left one, it should be coded as the more salient dimension. It follows from this that no difference should show between weak and strong right-footers in a valence judgment task in which they respond with their right or left foot; both groups should respond faster with the right foot (coded as +) to positive items (also coded as +), and with their left foot to negative items (-). The fact that only strong right-footers show a valence-by-left/right interaction indicates, however, that this account cannot explain the findings presented here; rather it is an indication that the degree of fluency of hand or foot plays a crucial role for the emergence (or non-emergence) of an association between valence and left/right. This observation fits also nicely with a recent study investigating positive and negative words and their association with vertical space, and which found compelling evidence for the role of specific experiences for such an association (Dudschig et al., 2015).

An interesting question following from this observation is whether individuals who are weak right-footers but strong right-handers should exhibit different patterns, depending on whether they respond with their hand or with their foot. Furthermore, if fluency of limbs lies at the core of the association between valence and left/right, individuals whose preferred hand and foot differ – that is, right-handers who prefer their left foot, and left-handers who prefer their right foot – should show opposing patterns, depending on the limb with which they respond in a valence judgment task. If the outcome for these participants does differ depending on the limb used for response, it would be interesting to see in non-motoric tasks whether individuals whose preferred hand and foot differ prefer the right or the left. In Experiment 3 reported by Casasanto (2009), participants indicated verbally whether they thought a positively associated entity should go in a box on the left or on the right. Employing such a paradigm could provide clues to whether individuals prefer the side of their dominant hand or of their dominant foot.

A final remark on the procedure employed in the present study: footedness of participants was made salient before the actual experiment. Participants first dribbled a small ball around obstacles, first with one foot, then with the other, in order to get a feeling which foot they might prefer. Afterward, they filled out the footedness questionnaire. In sum, participants’ attention was drawn to their footedness before the valence judgment task started. It would be interesting to see whether the salience of footedness had an impact on the observed results, or whether the association between valence and left/right for strong right- or left-footers would also show if participants’ attention is not drawn to their footedness, i.e., by not mentioning footedness before the experiment and filling out the footedness questionnaire afterward. For manual responses, we have observed valence-by-left/right interactions previously although no handedness questionnaire was given to participants before the actual experiment (de la Vega et al., 2012, Experiments 2 and 3). However, as footedness does not play such a prominent role as handedness in everyday life, we cannot conclude from these previous findings that an interaction between valence and foot responses shows if attention is not drawn to footedness. If no interaction shows when footedness is not salient, this would imply that rather than actual footedness, the *idea* of whether an individual thinks she is a (strong) right-footer should have an impact on responses. In this case, of course, fluency should not play any role at all for the emergence of such an interaction.

In sum, the present study not only extends existing literature by providing evidence for a compatibility effect between positive/negative and left vs. right foot for strong right-footers; it also hints at an influential role of the degree of fluency for the emergence of the association between valence and left/right. Future studies are needed to address whether the degree of handedness influences also bimanual responses to positive and negative items, and whether the self-conceptualization of an individual as (strong) right-footer or left-footer influences the valence-by-left/right association observed in a response times study.

## Conflict of Interest Statement

The authors declare that the research was conducted in the absence of any commercial or financial relationships that could be construed as a potential conflict of interest.

## Footnotes

1 Order of stimulus-response mapping was almost equally distributed among the strong right-footers, but not among the weak left-footers (this was due to the initial exclusion of participants due to left-footedness). An equal distribution of the order is, however, important, as participants become typically faster and less prone to errors during the second part of the experiment. A majority of participants for whom a mapping of positive to the right and of negative to the left comes in the second half of the experiment could consequently inflate the effect artificially, whereas a majority of participants responding in the second part to negative with the right and to positive with the left might deflate it. Indeed, the majority of the participants in the group of weak right-footers had started with a mapping of positive to the right and negative to the left, whereas eleven out of 21 participants in the group of strong right-footers had started with a mapping of negative to the right and positive to the left. To rule out that the observed effects are due to this unequal distribution, we ran the same analyses cutting off the last participants of each group to obtain an equal distribution. The strong right-handers (20 participants) showed basically the same results as described above, as did the remaining 12 weak right-footers [*F*_1_ < 1; *F*_2_(1,38) = 1.11, *p* = 0.30]. The latter result cannot be attributed to the small number of participants left in this analysis, as we see when cutting off strong right-footed participants to get the same sample size [*F*_1_(1,11) = 3.38, *p* = 0.09; *F*_2_(1,38) = 20.98, *p* < 0.001]. Related to this issue, and as a reviewer pointed out, one might expect differences between participants with regard to the compatibility effect depending on the order of stimulus-response mapping, that is, whether participants start with the fluent mapping (positive to the right, negative to the left) or with the non-fluent mapping (negative to the right, positive to the left). We ran an analysis including all 37 participants and the order of stimulus-response mapping (participants start with the mapping of positive to the right and negative to the left and proceed with the mapping of positive to the left and negative to the right, or the other way around) as a factor. In the by-participants analysis, the factor “order of stimulus-response mapping” did not interact with any variable (all *F*s < 1.80). In the by-items analysis, a significant three-way interaction between “order of stimulus-response mapping,” “valence,” and “foot” emerged [*F*_2_(1,38) = 13.19, *p* < 0.001]. This interaction can be attributed to the fact that the second part of the experiment is overall faster than the first block, which is due to the increased practice of participants. This practice enhances the compatibility effect for participants who had started with a non-fluent mapping and who go on with a fluent mapping in the second block, but obviously not for participants who had started with a non-fluent mapping. However, the compatibility effect described above cannot be attributed merely to a practice effect, as it is present already in the first part of the experiment [*F*_1_(1,35) = 4.71, *p* = 0.036; *F*_2_(1,38) = 70.51, *p* < 0.001]; albeit not, when analyzing weak and strong right-footers separately, for weak right-footers (both *F*s < 1).
